# Deep into Laboratory: An Artificial Intelligence Approach to Recommend Laboratory Tests

**DOI:** 10.3390/diagnostics11060990

**Published:** 2021-05-29

**Authors:** Md. Mohaimenul Islam, Tahmina Nasrin Poly, Hsuan-Chia Yang, Yu-Chuan (Jack) Li

**Affiliations:** 1 Graduate Institute of Biomedical Informatics, College of Medical Science and Technology, Taipei Medical University, Taipei 110, Taiwan; d610106004@tmu.edu.tw (M.M.I.); tahmina6969@gmail.com (T.N.P.); itpharmacist@gmail.com (H.-C.Y.); 2 International Center for Health Information Technology (ICHIT), Taipei Medical University, Taipei 110, Taiwan; 3 Research Center of Big Data and Meta-Analysis, Wan Fang Hospital, Taipei Medical University, Taipei 116, Taiwan; 4 Department of Dermatology, Wan Fang Hospital, Taipei 116, Taiwan; 5 TMU Research Center of Cancer Translational Medicine, Taipei Medical University, Taipei 110, Taiwan

**Keywords:** laboratory test, deep learning, artificial intelligence, recommendation system, clinical decision support system

## Abstract

Laboratory tests are performed to make effective clinical decisions. However, inappropriate laboratory test ordering hampers patient care and increases financial burden for healthcare. An automated laboratory test recommendation system can provide rapid and appropriate test selection, potentially improving the workflow to help physicians spend more time treating patients. The main objective of this study was to develop a deep learning-based automated system to recommend appropriate laboratory tests. A retrospective data collection was performed at the National Health Insurance database between 1 January 2013, and 31 December 2013. We included all prescriptions that had at least one laboratory test. A total of 1,463,837 prescriptions from 530,050 unique patients was included in our study. Of these patients, 296,541 were women (55.95%), the range of age was between 1 and 107 years. The deep learning (DL) model achieved a higher area under the receiver operating characteristics curve (AUROC micro = 0.98, and AUROC macro = 0.94). The findings of this study show that the DL model can accurately and efficiently identify laboratory tests. This model can be integrated into existing workflows to reduce under- and over-utilization problems.

## 1. Introduction

The laboratory test plays a vital role in patient care, accurate diagnosis, and treatment planning (approximately 60–70% of the clinical decision is made based on laboratory tests) [1,2]. Previous studies report that the rate of laboratory test ordering has been increased in general practice [3,4,5]; although they are frequently overused, misused, and even underused, which raises questions [6,7]. A higher proportion of laboratory test errors occur in the pre-analytical stage, leading to misdirected or delayed diagnosis, jeopardizing patients’ safety. An inappropriate laboratory test is not only associated with an increased risk of psychological and physical harm [8,9] but also causes unnecessary financial burden [10,11]. Unfortunately, there is no direct relationship between higher spending and healthcare benefits.

Inadequate knowledge of laboratory tests, availability of higher numbers of new tests, fear of diagnostic errors, and lack of education have been found to be associated with inappropriate laboratory testing [12,13]. Several factors also increased the rate of inappropriate laboratory testing, such as increasing patient satisfaction, fear of liability, and fee-for-service billing [10,14]. Existing strategies to reduce inappropriate tests and improve the efficiency of ordering clinical laboratory tests include feedback and reminder [15,16], clinical decision support tools [17], education [18], cost display [19], practice guidelines [20], and condition-specific algorithms [21]. The performance of these interventions varies, depending on how these interventions are applied.

Nowadays, the demand for automated laboratory recommendation systems for accurate and quicker diagnosis has been increased. An automated recommendation might improve the accuracy and efficiency of test orders and contribute to healthcare resource-saving. In this study, we developed a novel automated recommendation system-based deep neural network (DNN) that automatically predicts accurate and efficient laboratory tests using simple variables available in the electronic health records (EHRs). We evaluated DNN model performance and assessed the acceptability, generalizability, and interpretability by using standard guidelines.

## 2. Methods

### 2.1. Data Source

We collected data from the Taiwanese National Health Insurance Research and Development (NHIRD). The NHIRD stores all claims of medications, diagnoses, and laboratory work for 23 million people in Taiwan, which represents approximately 99.9% of the total population [22]. The NHIRD database includes patients’ demographic information, total number of prescriptions, date of prescriptions, number of medications, dosages, durations, and comorbidities history. The quality and completeness of the NHIRD database is excellent and it is used to conduct high-quality clinical researches [23,24]. The findings from these researches have been used in valuable clinical decision-making, patient care improvement, and policy-making. The Taipei Medical University (TMU) research ethical board approved this study. Participants’ consent was not required because the entire individual’s information was de-identified.

### 2.2. Data Collection and Study Populations

In this study, we collected patients’ demographics, medications, diseases, and laboratory information from a random sample of two million from the NHIRD between 1 January 2013, and 31 December 2013. We selected all prescriptions that contained at least one laboratory test.

### 2.3. Data Preprocessing

**Figure 1:**
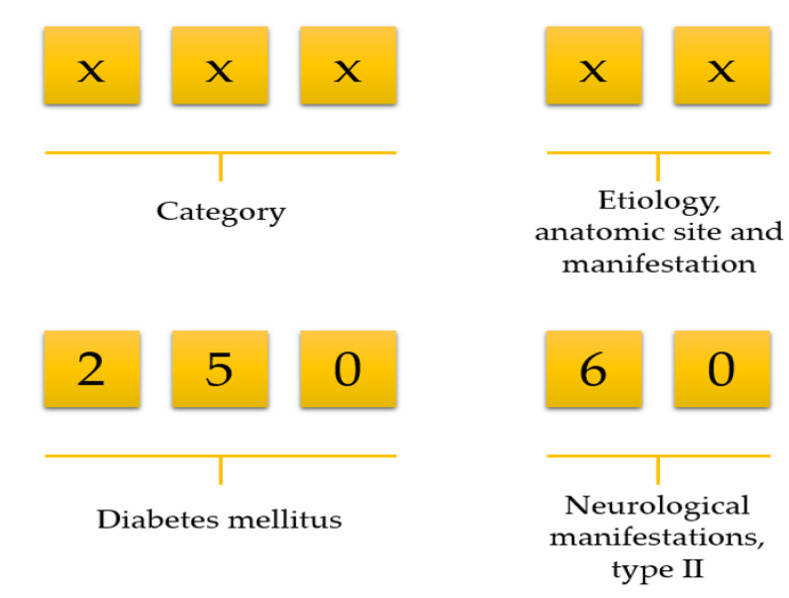
Selection of disease using the first three-digit code.

Data preprocessing consists of three steps: (1) data cleaning, (2) variable selection, and (3) matrix formation. NHIRD stores a vast amount of variables but all variables are not necessary for doing research. We first deleted unnecessary variables and kept only demographics, visit identification, visit date, date of birth, department identification, medications, diseases, and laboratory information. Then we selected patients who ordered at least one laboratory test on each visit. Afterwards, we calculated an age for all patients using their birth date and visit date information. There was no age limitation included in our work. The International Classification of Diseases, Ninth Revision, Clinical Modification (ICD-9-CM) code consists of a five-digit code; the first three digits describe the category, and the last two digits describe aetiology, anatomic site, and manifestation (Figure 1)**.** For example, ICD-9 of diabetes mellitus is 250. However, it has further been classified into several groups, such as 250.0: Diabetes mellitus without mention of complication, type II or unspecified type, not stated as uncontrolled, 250.1: Diabetes with ketoacidosis, and 250.10: Diabetes with ketoacidosis, type II or unspecified type, not stated as uncontrolled.

**Figure 2:**
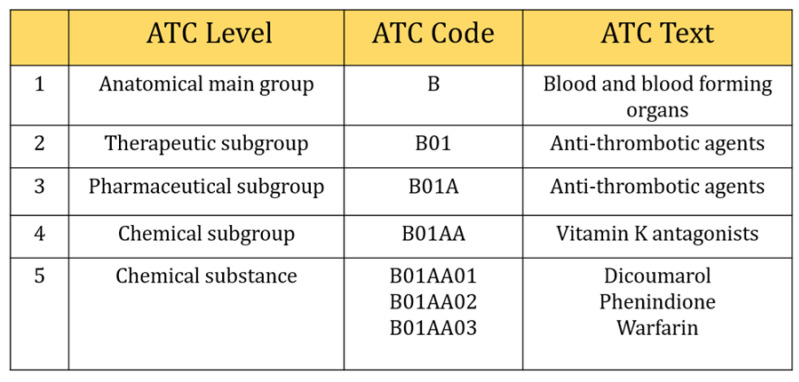
Selection of five digits ATC code.

However, we selected three digits of ICD-9-CM, including the V-code (supplementary classification of factors influencing health status and contact with health services) classification to retrieved comorbidities information. They were usually distributed from 001~999 and V01~V82. In the case of Anatomical Therapeutic Chemical Classification (ATC), five characters of ATC code were considered in this study (Figure 2), e.g., five-digit ATC code: A10BA (Biguanides), blood glucose lowering drugs, including all drugs like A10BA01 (phenformin), A10BA02 (metformin), and A10BA03 (buformin). Also, seven characters (e.g., A10BA02) were considered as chemical substances even though five characters were used to describe chemical substances and all drugs included in this group were prescribed for almost the same purpose. There were 654 different types of laboratory tests ordered during the study period. We calculated the frequency of laboratory tests and selected those tests that contributed at least 150 prescriptions (0.001 per cent) during our study period. This is because a deep learning algorithm needs sufficient data to train the model, otherwise, it can perform poorly. Finally, 315 types of tests were included in our study.

**Figure 3:**
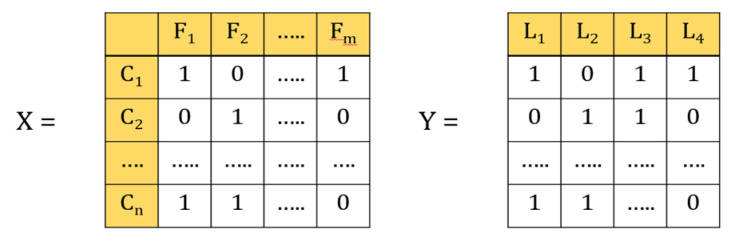
Illustrations of multi-label classification. X is the data set in which feature vectors represent patients C_1_–C_n_; *n* is the number of patients; F_1_–F_m_ are variables; m is the number of variables. Y is the label vector; L_1_–L_4_ represents the number of lab tests.

In the multi-label classification, each instance belongs to several labels simultaneously (Figure 3). In our case, each laboratory test represents a label, and a patient can have different types of laboratory test at the same time. For example, if the data contains four labels, such as glucose, HbA1c, urine, and creatinine, then that patient can have 24 types of outcomes, such as only glucose, only HbA1c, only urine, only creatinine, or all glucose, HbA1c, urine, and creatinine, or glucose and HbA1c, and so forth and so on.

### 2.4. Model Building

**Figure 4:**
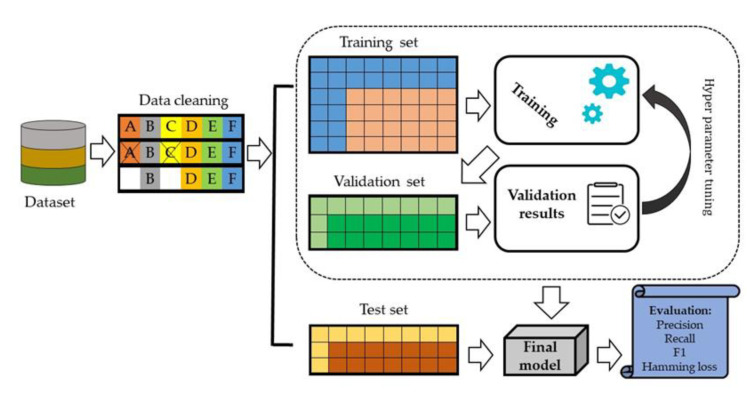
An architecture of the proposed deep learning model.

We split data set into 70% training set and 30% testing set. In the internal validation, 20% of the data was randomly selected from the training set and evaluated against model performance (Figure 4).

The DNN model was developed to train all variables and the model was assessed using the validation set to predict laboratory tests. DNN is a high-performing algorithm in which an artificial neural network consists of multiple layers. The input of DNN moves through the layers calculating the probability of each output. We used three hidden layers in our model. The activation function ReLU was used in the hidden layers and Sigmoid was used in the output layer. The activation function is an integral part of a neural network that is often known for non-linearity, i.e., describing the input and output relations in a non-linear way. However, the non-linearity element allows for higher flexibility and makes a complex function during the whole model learning process. It helps to speed up the whole learning process. Several activation functions, such as sigmoid and ReLU, are commonly used in practice.

(a) Sigmoid Function:

This takes a real-value input and converts it to a range between 0 and 1. The sigmoid function is defined as follows:

(1) $$\sigma \left(x\right)=\frac{1}{1+e^{-x}}$$

Here it is clear that it will convert output between 0 and 1 when the input varies in (−∞,∞). A neuron can use the sigmoid for computing the nonlinear function σ (y=wx+b). If y=wx+b is very large and positive, then $e^{-y}\to 0$, so σ(y)→1, while y=wx+b is very large and negative $e^{-y}\to \infty$, so σ(y)→0.

(b) ReLU: This stands for Rectified Linear Unit and it takes a real input variable and thresholds it at zero (replacing native values with zero). The ReLU function is defined as follows:

(2) f=max(0, x)

**Figure 5:**
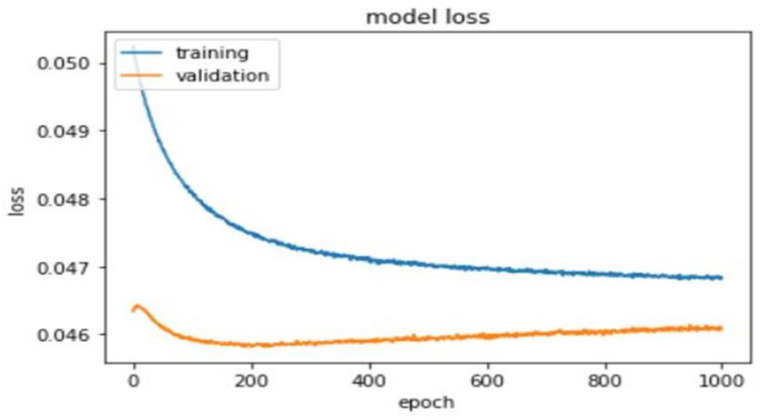
Training and validation loss of the deep learning model.

A total of 1000 epochs was utilized to calculate training and validation loss (Figure 5).

### 2.5. Evaluation Matrices

We evaluated the performance of our model on the validations set for laboratory tests with recommendations using the following metrics.

*Micro-AUC*: This is an averaging of the prediction matrix. *S*_micro_ is the set of correct quadruples. The equation of assessing Micro-AUC is as follows:

(3) $$Micro-AUC=\frac{\left|S_{micro}\right|}{\left(\sum_{i=1}^{m}\left|Y_{i.}^{+}\right|)\cdot (\sum_{i=1}^{m}\left|\;Y_{i.}^{-}\right|\right)}$$

(4) $$S_{micro}=\{\left(a,b,i,j\right)|\left(a,b\right)\in Y_{.i}^{+}\times Y_{.j}^{-},\;f_{i}\left(x_{a}\right)\geq f_{j}\left(x_{b}\right)\}$$

*Macro-AUC* = This is an averaging of each label. $S_{macro}$ is the set of correctly ordered instance pairs on each label. The equation for assessing macro-AUC is as follows:

(5) $$Macro-AUC=\frac{1}{l}\;\overset{l}{\underset{j=1}{\sum }}\frac{\left|S_{macro}^{j}\right|}{\left|Y_{.j}^{+}\right|\left|Y_{.J}^{-}\right|}$$

(6) $$S_{macro}^{j}=\{\left(a,b\right)\in Y_{.j}^{+}\times Y_{.j}^{-}|f_{i}\left(x_{a}\right)\geq f_{i}\left(x_{b}\right)\}$$

*Micro-F1*: This is an averaging of the prediction matrix. The equation is as follows:

(7) $$Micro-F1=\frac{2\sum_{j=1}^{l}\sum_{i=1}^{m}y_{ij}h_{ij}}{\sum_{j=1}^{l}\sum_{i=1}^{l}y_{ij}+\sum_{j=1}^{l}\sum_{i=1}^{m}h_{ij}}$$

*Macro-F1*: This is an averaging of each label. The equation for assessing macro-F1 is as follows:

(8) $$Macro-F1=\frac{1}{l}\;\overset{l}{\underset{j=1}{\sum }}\frac{2\sum_{i=1}^{m}y_{ij}h_{ij}}{\sum_{i=1}^{m}y_{ij}+\sum_{i=1}^{m}h_{ij}}$$

*Average Precision*: Average precision summarizes the fraction of relevant labels ranked higher than one other relevant label. The equation is as follows:

(9) $$Average\;Precision=\frac{1}{m}\overset{m}{\underset{i=1}{\sum }}\frac{1}{\left|Y_{i.}^{+}\right|}\;\underset{j\in Y_{i.}^{+}}{\sum }\frac{\left|S_{precision}^{i,j}\right|}{rank_{F}\left(x_{i}\;,\;j\right)}$$

(10) $$S_{precision}^{ij}=\{k\in Y_{i.}^{+}|rank_{F}\left(x_{i\;},k\right)\leq rank_{F}\left(x_{i},\;j\right)\}$$

*Hamming Loss (HL)*: This is the average symmetric difference between the set of true labels and the set of predicted labels of the data set. The equation is as follows:

(11) $$Hloss\;\left(H\right)=\frac{1}{ml}\;\overset{m}{\underset{i=1}{\sum }}\overset{l}{\underset{j-1}{\sum }}⟦h_{ij}\neq y_{ij}⟧$$

*HL* value ranges from 0 to 1. A lesser value of hamming loss indicates a better classifier. The analyses were performed using Python (version 3.7.3; https://www.python.org (accessed on 1 January 2020)).

## 3. Results

### 3.1. Data Descriptions

We collected all patients’ data retrospectively who visited a hospital between 1 January 2013 and 31 December 2013. A total of 536,015 (26.80%) patients were ordered laboratory tests, consisting of 1,491,564 prescriptions. After excluding infrequent laboratory test orders, we finally considered 315 different types of laboratory tests from 530,050 patients with 1,463,837 prescriptions to develop our prediction model. The number of female patients was higher than male patients (44.05% [233,509/530,050] vs. 55.95% [296,541/530,050] and the age of patients ranged from 1 to 107 years.

### 3.2. Model Performance

**Figure 6:**
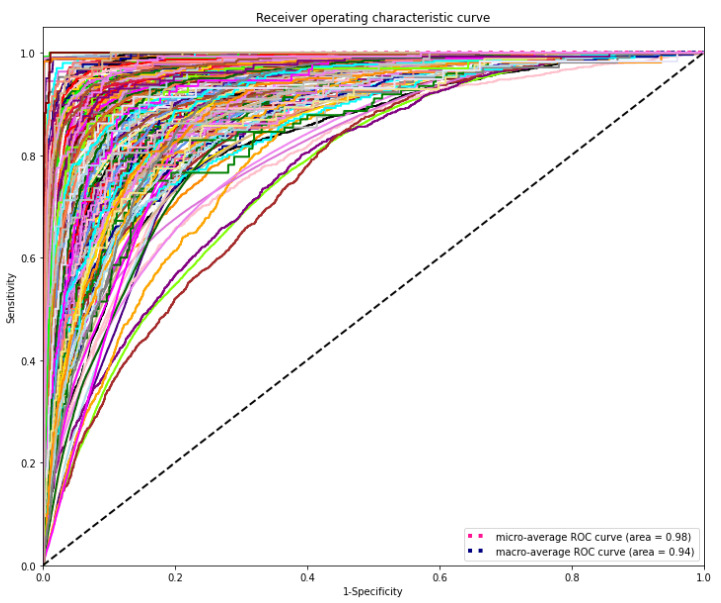
Receiver operating characteristic (ROC) curves of the deep learning model for predicting laboratory tests. The range of AUROC was between 0.76 and 1 (Supplementary Table S1). The highest AUROC 1 was HIV viral loading test and the lowest AUROC 0.76 was for CBC-Ⅲ (WBC, RBC, HB, HCT, and MCV).

The DL model demonstrated the highest discriminating power for all diagnostic laboratory tests using minimal variables from EHR, yielding a mean AUROC _micro_ of 0.98 and AUROC _macro_ of 0.94, respectively (Figure 6).

Table 1 presents the performance of the DL model for the prediction of laboratory tests in varying cut-off points. Our prediction model showed the highest recall 0.96 when the cut-off value 0.01 was considered. However, precision, recall, and F1 score value were almost similar at the cut-off value 0.30 and 0.35. Hamming loss significantly was going down while increasing cut-off value reached 0.011 when cut-off value 0.4 was considered.

### 3.3. Distribution of AUROC

**Figure 7:**
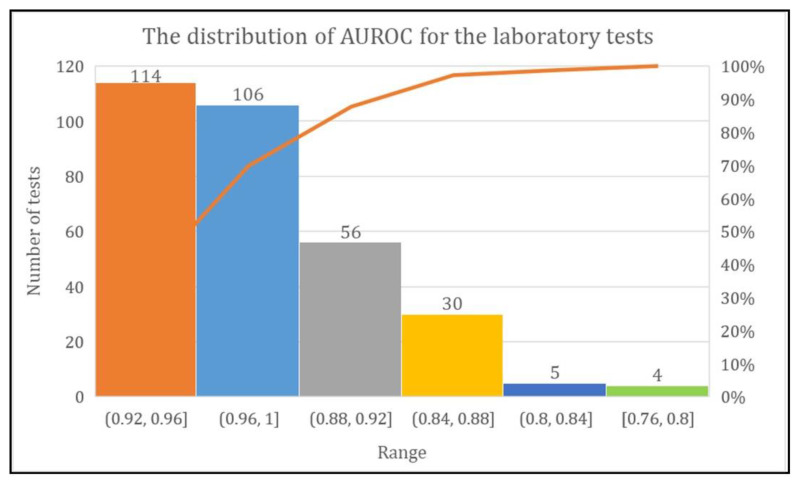
Total number of laboratory tests with their AUROC range.

The DL model achieved more than 80% AUROC for the prediction of every laboratory test except two tests named urine biochemistry examination and CBC-Ⅲ. The range of AUROC was between 0.76 and 1. However, the DL model achieved AUROC of 0.92–0.96 for 114 laboratory tests, 0.96–1 for 106 laboratory tests, and 0.88–0.92, 0.84–0.88, 0.80–0.84, and 0.76–0.80 for 56, 30, five, and four laboratory tests, respectively (Figure 7).

### 3.4. Evaluation

**Figure 8:**
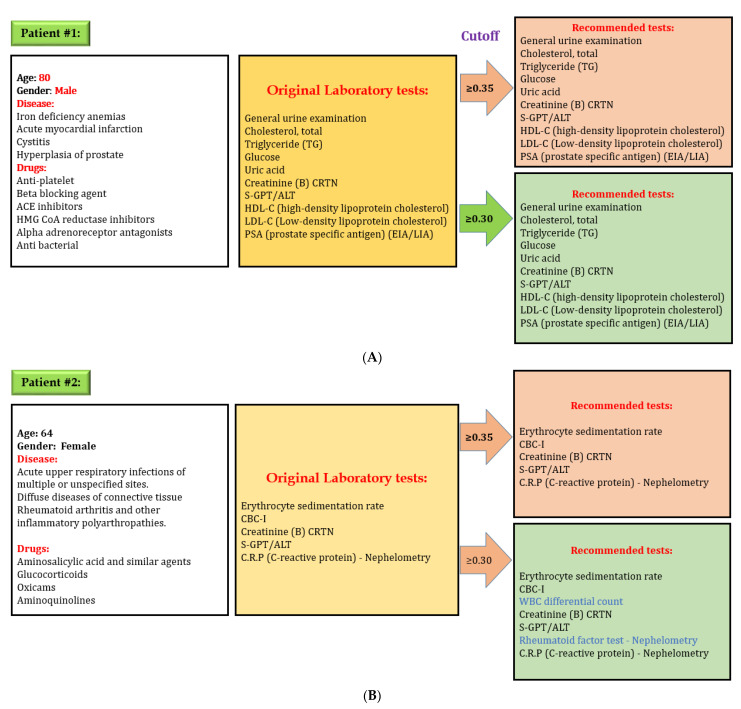
Evaluation of the performance of the deep learning model for predicting laboratory tests, (**A**) system always give exact recommendation for laboratory tests with a cut off value 0.35, (**B**) system might give extra recommendation of laboratory tests with a cut off value 0.30; however all laboratory recommendations are correct. Physicians can adjust cut-off value if additional tests are required. Decreasing cut-off value will increase the number of laboratory tests recommendation.

After developing the DL model to predict laboratory tests, we evaluated the effectiveness of our model. We used the “lab test online” for evaluating laboratory testing appropriateness, which contains all information, including disease and various laboratory tests in detail. We selected some random cases from the testing set for preliminary evaluations to evaluate our model performance. Figure 8 shows one example of evaluation of the DL model. Here, an 80-year-old male patient had four diseases: iron deficiency anemia, acute myocardial infarction, cystitis, and hyperplasia of the prostate. The physician prescribed several medications for his problems and ordered 12 laboratory tests based on the patient’s physical condition. Our model predicted the same laboratory tests while considering cut-off value 0.35 and 0.30. However, our model recommends two additional laboratory tests while considering cut-off value 0.30 for a second patient. There are credible reasons for these two tests in this situation because a patient with rheumatoid arthritis and other inflammatory polyarthropathies needs to check WBC differential count on a regular basis. Rheumatoid factor test–Nephelometry is often used when a patient is suspected of having a kidney problem. The physician may adjust cut-off value when more tests are needed because the laboratory test depends on the patient’s specific condition.

## 4. Discussion

### 4.1. Main Findings

Our primary objective was to reduce inappropriate laboratory test ordering by developing a DL model based on simple variables available in the EHR. This study represents the first investigation using the DL model to develop and internally validate a diagnostic laboratory tests prediction model. The DL model has great potential for predicting events and recognizing data patterns used in many healthcare industry areas. Our findings are fascinating since the DL model showed an excellent caliber for predicting laboratory tests (AUROC micro = 0.98 and AUROC macro = 0.94). The range of AUROC was between 0.76 and 1. This study suggests considering a threshold between 0.30 and 0.35 to achieve optimal laboratory test recommendations. However, the recall is higher (more than 0.95) when the threshold is 0.01, which would recommend all possible laboratory tests in the recommendation system. All these laboratory tests have credible reasons for the recommendation, and there is no issue for false negatives.

Defining appropriate laboratory test orders is not an easy task and is influenced by several factors, but the findings of our study show that the DL model can predict appropriate laboratory tests and ensure personalized treatment. Only four features, namely patients’ age, gender, diseases, and drugs history, are synthesized through the DL model to make vigorous and accurate predictions. Various applications and clinical contexts might need a different level of acceptability, therefore, this study gives all possible recommendations while choosing a potential target recall close to 96% (when our model predicts laboratory tests result as correct, the goal was for it to be right 96% of the time). Moreover, the results of our study suggest that our model may be implemented in real-world clinical settings because it had only a 4% false-negative prediction. The recommendation system based on the DL prediction model using clinical data provided would assist physicians in decision-making where long lists of laboratory tests are needed but not replace them. One flexible feature for utilizing the current model is that physicians could use a different threshold to get the varying level of laboratory test recommendations based on patient characteristics. For example, S-GOT/AST, and S-GPT/ALT are not essential to perform on every patient with hypertension. Nevertheless, the physician can increase the cut-off value to get more recommendations and order these tests if they suspect any symptoms of kidney problem.

### 4.2. Current Research Gap and Possibility

Laboratory testing is a crucial part of healthcare for screening, diagnosing, treating, and monitoring disease progression. The use of laboratory tests is growing fast, therefore, selecting appropriate testing is important to improve patient safety by reducing diagnosis error [25,26]. Studies have pointed out the higher rate of the under- and over-utilization problem, indicating a perceivable gap for quality improvement [26,27,28]. There is inadequate evidence and a research gap to support appropriate laboratory test ordering. Selecting appropriate tests for an individual patient is challenging for physicians. The probable influence of hidden interaction between laboratory tests has been overlooked [29]. Physicians usually use their medical knowledge, judgment, and experience to ensure potential tests for patient diagnosis and management. Furthermore, physicians rely on computerized provider order entry (CPOE) rule-based decision support tools to order laboratory tests. Actual benefits of these systems for the appropriateness of laboratory test ordering remain unclear [30]. Utilizing deep learning models for laboratory data integration and analyses will offer an enormous opportunity to improve diagnostic value [31]. As the number of diagnostic tests is steadily increasing, and their ordering pattern has been changing over time, it is essential to recognize and extract valuable information from numerous laboratory data that may help clinical practice. A deep learning model can forge a practical path of ensuring appropriate laboratory test utilization based on previously unrecognised patterns.

### 4.3. Clinical Implications

Inappropriate laboratory test ordering is a neglected issue which hampers patient safety and unnecessarily increases costs [12]. Inappropriate laboratory test ordering appears to be a common problem across all high-resource healthcare settings [32,33]. This current study’s findings recommend a more wide-scale implementation of this system on national-level hospitals to reduce over- and under-utilization problems. Our recommendation model is rigorous and developed based on clinical practice data, therefore, it can be implemented at international level by adjusting thresholds. Various machine-learning models are available for laboratory test results, but no study focuses on laboratory test ordering. Over-utilization of laboratory tests has been reported in many studies [34,35]. However, in our current study, we focused extensively on pre-analytical laboratory test ordering error and developed a DL model that recognizes laboratory data patterns and provides personalized recommendations. Recently, Wright et al. [36] developed association rule mining for identifying a possible association between disease and laboratory tests results using 272,749 diseases, and 11,801,068 laboratory results. However, their automated technique for predicting test ordering has significant limitations, while our model predicted laboratory tests with a convincing performance.

Ideally, this DL-based clinical laboratory test ordering system may be integrated into the CPOE, and it will work in the background providing real-time, personalized laboratory test recommendations based on the patients’ available clinical histories. Consequently, physicians do not need to consider the complex and hidden interconnection between laboratory tests, guidelines, cost display, and fear of missing orders. Indeed, the system can improve clinical practice. Another benefit of using this model is collecting more patient data over time and improving its predictive ability. Our model only used four types of variables. These clinical variables are routinely recorded during visits to the hospital, therefore, these variables are always available in the EHR. Moreover, our model was designed to handle multiple outcomes at the same time.

### 4.4. Limitations

Our study has several limitations. First, we did not consider the temporal dimension in our current study. However, few laboratory tests depend on the temporal dimension. Second, our study did not include procedure code. However, some laboratory tests are connected to the procedures. Third, we did not externally validate our findings. Therefore, our findings might vary with other datasets. Although, we internally validated our model.

### 4.5. Future Prospects

The higher recall and precision at varying cut-off value levels suggest that our model can be integrated into the EHR to recommend personalized laboratory tests. We anticipate that the role of a DL model for suggesting laboratory tests will become a favoured and helpful tool in the near future. This approach will be a new pathway of ensuring improved patient care. In future, we have a plan to include more variables, such as procedure codes, to predict laboratory tests. This is because some laboratory tests fully depend on procedures. Moreover, we plan to add more data to improve our current study’s performance because the DL model works even better when the data volume is even higher. We will try to cover all laboratory tests as well as laboratory procedures in the future model. Furthermore, inpatients’ data and patients’ clinical notes will be considered to make our recommendation system even better.

## 5. Conclusions

We developed and validated the DNN model for recommending personalized laboratory tests using simple variables available in the EHR. The findings of our study show that this model has immense potential to predict appropriate laboratory tests, and help to reduce over- and under-utilization. Therefore, integrating this model into electronic health records systems may facilitate optimal test selection and reduce under- and over-utilization problems. However, the DL model is often referred to as a “black box” due to the lack of interpretability of findings [37,38]. Future studies are needed to evaluate the effectiveness of DL to predict laboratory tests.

## Figures and Tables

**Table 1:** The performance of DL model based on varying cut-offs for clinical laboratory test prediction.

Cut-Off	Precision	Recall	F1 Score	Hamming Loss
≥0.01	0.24	0.96	0.37	0.082
≥0.05	0.32	0.89	0.46	0.038
≥0.10	0.39	0.83	0.52	0.025
≥0.15	0.44	0.76	0.55	0.019
≥0.20	0.48	0.72	0.56	0.016
≥0.25	0.52	0.66	0.57	0.014
≥0.30	0.55	0.61	0.57	0.013
≥0.35	0.57	0.57	0.55	0.012
≥0.40	0.61	0.51	0.54	0.011
≥0.45	0.63	0.47	0.51	0.011
≥0.50	0.65	0.42	0.48	0.011

## Data Availability

Not applicable.

## Supplementary Materials

The following are available online at https://www.mdpi.com/article/10.3390/diagnostics11060990/s1.
