# Supplementary material for: Deep into Laboratory: An Artificial Intelligence Approach to Recommend Laboratory Tests
Source: Diagnostics (Basel). 2021 May 29;11(6):990. doi: 10.3390/diagnostics11060990 (PMC8227070; doi:10.3390/diagnostics11060990)
Supplement: Supplementary file 1 [file diagnostics-11-00990-s001.zip › diagnostics-1181645-supplementary.pdf]

Supplementary Table S1: The area under receiver operating curve (AUROC) value for individual test.

Test Name	AUROC
pH	0.92
Urine protein	0.86
Urine sugar	0.93
Ketone body	0.96
Urine Sediments	0.83
General urine examination (including protein, sugar, urinary bilirubin, bilirubin, urine sediment, specific gravity, color, turbidity, leukocyte ester?, occult blood, pH, and ketone bodies)	0.88
Urine biochemistry examination	0.77
Urine-osmolality test	0.93
Pregnancy test -Latex agglutination	0.98
Pregnancy test -EIA	0.99
Occult blood(chemical method)	0.88
Neutral fat stain	0.97
Fatty acid stain	0.96
Stool routine	0.9
Parasite Concentration Method	0.91
Stool WBC	0.96
R.B.C	0.9
W.B.C	0.85

Hemoglobin (Hb)	0.86
Hematocrit (Hct)	0.92
E.S.R.(Erythrocyte sedimentation rate)	0.97
Platelet count	0.86
Reticulocyte count	0.93
RBC morphology	0.89
Eosinophil count	0.96
CBC-I(WBC,RBC,HB,HCT,PLATELET COUNT,MCV,MCH,MCHC)	0.89
CBC-II(WBC、RBC、Hb、Hct、MCV、MCH、MCHC）	0.8
WBC differential count	0.93
Blood routine	0.78
Bleeding time (Duke method)	0.94
Bleeding time (Ivy method)	0.94
Clotting time, coagulation time(Lee-White method)	0.96
Fibrinogen (quantitative)	0.9
Fibrinogen (Nephelometry)	0.88
Prothrombin time	0.94
Hb electrophoresis	0.92
APTT (activated partial thromboplastin time)	0.94
FDP (fibrin degradation product) -quantitative	0.92
Blood osmolality	0.95
D dimer test	0.9

CBC-III(WBC、RBC、HB、HCT、MCV)	0.76
MCH	0.95
MCHC	0.95
Protein S	0.91
Lupus anticoagulant test	0.95
MCV	0.9
Insulin serum test	0.86
Cystatin C	0.97
Cholestero1, total	0.88
BUN, blood urea nitrogen	0.85
Urea-N (urine)	0.97
Triglyceride (TG)	0.87
Glucose	0.92
HbA1c (Hemoglobin A1c)	0.88
T3 Uptake Ratio	0.92
T4	0.93
Ca (Calcium)	0.93
P (Phosphorus)	0.97
Uric acid	0.87
Creatine	0.81
Creatinine (B) CRTN	0.82
Creatinine (U) CRTN	0.86

Amylase (B)	0.95
Fe (Iron-bound)	0.95
Na (Sodium)	0.94
K(Potassium)	0.93
Cl (Chloride)	0.89
Carbon dioxide	0.98
S-GOT/AST	0.84
S-GPT/ALT	0.81
Alkaline phosphatase	0.93
Bilirubin total	0.9
Bilirubin direct	0.88
r-GT(r-glutamyl transferase)	0.86
CPK(Creatine-phospho-kinase)	0.91
LDH(Lactic dehydrogenase)	0.87
TIBC(Total Iron Binding Capacity)	0.95
Blood ammonia	0.96
Albumin	0.94
Globulin	0.94
Total protein	0.88
Blood gas analysis	0.97
HDL-C (high-density lipoprotein cholesterol)	0.85
LDL-C (Low-density lipoprotein cholesterol)	0.87
Mg (Magnesium)	0.91

Cu (Copper)	0.91
Zn (Zinc)	0.93
VMA (Vanillyle-mandelic-acid) (quantitate)	0.91
Lactic Acid (lactate)	0.97
CPK isoenzyme	0.94
Lipase	0.96
protein electrophoresis	0.93
CK-MB (Creatine phosphokinase-MB)	0.96
Catecholamine	0.92
Stone analysis	0.97
Transcutaneous bilirubin	0.99
Troponin T	0.95
ADA	0.95
Insulin (EIA/LIA)	0.89
Progesterone (EIA/LIA)	0.98
Free T4 (EIA/LIA)	0.92
Free T3 (EIA/LIA)	0.94
GH (Growth hormone), EIA/LIA	0.91
TSH(EIA/LIA)	0.92
Aldosterone (EIA/LIA)	0.91
ACTH (EIA/LIA)	0.86
Prolactin (PRL), EIA/LIA	0.96
FSH (EIA/LIA)	0.97

LH (EIA/LIA)	0.97
Estradiol (E2), EIA/LIA	0.97
VIT-B12 (EIA/LIA)	0.94
Folic acid (EIA/LIA)	0.94
Stool occult blood (iFOB)	0.91
Serum ketone body (quantitative)	0.95
Aluminum (Al)	0.98
Zinc(Zn)(AA method)	0.94
Carbamazepine	0.98
Diphenylhydantoin	0.98
Theophylline	0.93
Valproicacid	0.99
Digoxin	0.95
Li(Lithium)	0.99
Therapeutic drug monitoring - cyclosporine	1
Therapeutic drug monitoring - diazepam (valium)	0.98
Benzodiazepine(quantitative)	0.97
Therapeutic drug monitoring - TCA (tricyclic antidepressant)	0.97
Therapeutic drug monitoring-FK-506	1
Sirolimus	1
Drug test (qualitative)	0.96
Barbiturate	0.97
Acetaminophen	0.98

Carboxyl hemoglobin	0.97
Ethyl alcohol	0.97
Amphetamine (EIA)	0.97
Morphine (EIA)	0.97
Cocaine (EIA)	0.97
Cannabinoids (EIA)	0.98
Ethanol-drunk not driving	0.97
Ethanol-DUI (Driving under the influence)	0.97
A.B.AB.O blood grouping	0.96
Cross matching test	0.98
RH(D) type inspection	0.97
Antibody screening	0.98
Antibody identification	0.97
RPR/VDRL test	0.92
ASLO, anti-streptolysin-O test -Nephelometry	0.91
AFP α-fetoprotein（EIA/LIA）	0.93
Cold hemagglutinin	0.97
Rheumatoid factor test - Latex agglutination	0.95
Rheumatoid factor test - PHA(passive hem-agglutination)	0.97
Rheumatoid factor test - Nephelometry	0.96
C.R.P (C-reactive protein) - Latex agglutination	0.87
C.R.P (C-reactive protein) - Macinni immunodiffusion	0.88

C.R.P (C-reactive protein) - Nephelometry	0.93
TPPA/TPHA test	0.93
CEA（EIA/LIA）	0.92
β-HCG（EIA/LIA）	0.96
IgG - Nephelometry	0.96
IgA - Nephelometry	0.96
IgM - Nephelometry	0.94
IgE-Nephelometry	0.97
C3 - Nephelometry	0.97
C4 -Nephelometry	0.97
Haptoglobin-Nephelometry	0.95
Ceruloplasmin-Nephelometry	0.95
β2-microglobulin	0.94
ANA (antinuclear antibody) IFA	0.94
AMIA, anti-microsomal antibody	0.96
AMA, anti-mitochondrial antibody	0.95
ASMA, anti-smooth muscle antibody	0.95
APCA, anti-parietal cell antibody	0.98
Anti-DNA	0.98
Myoglobin	0.93
Cryoglobulin	0.94
Anti-ENA test (qualitative)	0.97

Anti-ENA-Ro/La Ab	0.97
Intercellular substance antibody	0.94
Thyroglobulin antibody	0.97
Cryptococcus antigen	0.94
Lymphocyte surface marker-Infectious disease	1
SCC (EIA/LIA)	0.92
PSA(prostate specific antigen) (EIA/LIA)	0.96
Direct Coombs' polyspecific test	0.95
Indirect Coombs' polyspecific test	0.95
Immunoelectrophoresis	0.94
CH50	0.99
Chlamydia Ab	0.91
Pre-albumin	0.97
Micro-albumin (Nephelometry)	0.87
Ferritin (EIA/LIA)	0.95
C-Reactive protein (Capillary ppt)	0.9
Anti-β2-glycoprotein-I Ab	0.97
Anti-TPO Ab	0.96
IgG4	0.92
Anti Jo-1 antibody	0.96
Immunoglobulin κ/λ	0.99
TNF	0.99
ANCA (Anti-neutrophil cytoplasmic Ab)	0.96

Anti-ENA, Sm/RNP Ab	0.98
Anti-ENA,Scl-70 Ab	0.97
HLA-ABC single antigen	0.93
Acetylcholine receptor antibody	0.97
DNA qualitative amplification test	0.93
DNA quantitative amplification test	0.98
RNA quantitative amplification test	0.98
Legionella pneumophila Ag (urine)	0.99
Pro calcitonin (PCT)	0.95
Pro-BNP/(BNP)	0.95
HLA-B 1502 gene typing	0.95
TDM - Everolimus	1
Free PSA (EIA/LIA)	0.97
Anti-cyclic citrullinated peptide	0.99
Sputum Routine (Include Crystal, Parasite)	0.95
Microscopic examination of excreta, exudates and secretions	0.94
Bacterial culture identification inspection	0.93
Acid-fast culture	0.96
Acid fast bacteria culture	0.96
Sensitivity test of acid-fast	0.96
Blood culture	0.98
Helicobacter pylori test (Clo test)	0.97

Bacteria MIC test (1 species)	0.92
Bacterial minimal inhibition concentration rapid detection	0.94
Acid-resistant concentrated smear stain inspection	0.96
Acid-fast bacteria culture (limited to the simultaneous use of solid medium and liquid culture system with automatic detection function)	0.96
Isolation and identification of virus	0.95
Cytomegalovirus CMV EIA	0.88
Herpes simplex virus CF HSV Ab (each)	0.92
Influenza A CF Ab	0.98
Influenza B CF Ab	0.98
ROTA VIRUS Ag	0.98
HBsAg (EIA/LIA)	0.89
Anti HBs	0.87
HBeAg (EIA/LIA)	0.96
Anti-HBe（EIA/LIA）	0.96
Anti-HBc（EIA/LIA）	0.87
Anti-HBc IgM (EIA/LIA)	0.9
Anti-HAV IgM（EIA/LIA）	0.93
Anti HAV	0.92
EB VCA IgG, IgM, IgA, IFA METHOD, EACH	0.92
EBNA Ab	0.93

Cytomegalic virus IgM Ab	0.88
ANTI-HIV TEST (EIA/LIA)	0.9
ANTI-HIV TEST PA	0.9
HCV Ab(EIA/LIA)	0.89
Herpes simplex virus IgM Ab	0.9
HSV-IgG	0.96
HIV viral load test	1
Body fluid cytology	0.92
Needle aspiration cytology	0.93
Pap's smear (Cytology 部分)	0.99
Fluid cytology plus cell block	0.94
Blood smear interpretation	0.97
Semen analysis	0.98
Ascetic fluid analysis	0.97
Pleural fluid analysis	0.97
CSF	0.95
Pus or discharge analysis	0.99
Synovial fluid analysis-Routine	0.98
Synovial fluid analysis-Crystal exam	0.98
Plasma free Ca++	0.9
Oral glucose tolerance test	0.9
Somatomedin C (insulin-like growth factor 1)	0.96

T3 (Triiodothyronine)	0.93
T4 (Thyroxine)	0.95
TSH (thyroid stimulating hormone)	0.92
FTI	0.94
Cortisol	0.86
IgE	0.98
PRL (prolactin)	0.96
FSH (follicle stimulating hormone)	0.97
LH (luteinizing hormone)	0.97
Insulin	0.91
Ferritin	0.96
Estradiol (E2)	0.97
Testosterone	0.96
Intact PTH	0.97
Free T4	0.93
Vitamin B12	0.94
Folic acid	0.94
Aldosterone	0.9
PRA (Plasma renin activity)	0.91
HBsAg	0.89
Anti-HBs	0.94
HBeAg	0.97
Anti-HBe	0.98

Cyclosporine-A	0.98
TIBC	0.95
AFP	0.95
CEA	0.95
PSA(prostate specific antigen)	0.97
CA-125	0.94
CA-153	0.98
CA-199	0.94
Anti-SCC	0.93
Ab-thyroglobulin	0.97
Thyroglobulin	0.98
Micro albumin	0.89
Ab-TSH receptor	0.98
Anti-ds DNA	0.98
ACTH	0.87
TPA	0.92
DHEA-SO4	0.96
Free PSA	0.98
Nasal smear	1
Anti-cardiolopin—IgG	0.98
Allergen test(qualitative)	0.98
Specific Allergen Test	0.98
ECP Test	0.98

Anti-phospholipid antibody—IgG	0.97
Anti-cardiolopin—IgM	0.98
Anti-phospholipid antibody—IgM	0.99
13C-urea breath test for Helicobacter pylori infection	0.98
Vomit occult blood test	0.92
Saliva production	0.99
Pad test	0.98
